# Novel Therapeutic Target for Prevention of Neurodegenerative Diseases: Modulation of Neuroinflammation with Sig-1R Ligands

**DOI:** 10.3390/biom12030363

**Published:** 2022-02-25

**Authors:** Ferenc Bogár, Lívia Fülöp, Botond Penke

**Affiliations:** 1 MTA-SZTE Biomimetic Systems Research Group, Eötvös Loránd Research Network (ELKH), Dóm Square 8, H-6720 Szeged, Hungary; bogar@sol.cc.u-szeged.hu; 2 Department of Medical Chemistry, University of Szeged, Dóm Square 8, H-6720 Szeged, Hungary; fulop.livia@med.u-szeged.hu

**Keywords:** neurodegenerative diseases, Alzheimer’s disease, ER-stress, neuroinflammation, microglia, astrocytes, cytokines, sigma-1 receptor ligands, non-steroid anti-inflammatory drugs

## Abstract

Neurodegenerative diseases (NDDs) are characterized by progressive deterioration of the structure and function of cells and their networks in the nervous system. There are currently no drugs or other treatments that can stop the progression of NDDs. NDDs have many similarities and common pathways, e.g., formation of misfolded amyloid proteins, intra- and extracellular amyloid deposits, and chronic inflammation. Initially, the inflammation process has a cytoprotective function; however, an elevated and prolonged immune response has damaging effects and causes cell death. Neuroinflammation has been a target of drug development for treating and curing NDDs. Treatment of different NDDs with non-steroid anti-inflammatory drugs (NSAIDs) has failed or has given inconsistent results. The use of NSAIDs in diagnosed Alzheimer’s disease is currently not recommended. Sigma-1 receptor (Sig-1R) is a novel target for NDD drug development. Sig-1R plays a key role in cellular stress signaling, and it regulates endoplasmic reticulum stress and unfolded protein response. Activation of Sig-1R provides neuroprotection in cell cultures and animal studies. Clinical trials demonstrated that several Sig-1R agonists (pridopidine, ANAVEX3-71, fluvoxamine, dextrometorphan) and their combinations have a neuroprotective effect and slow down the progression of distinct NDDs.

## 1. Introduction

Neurodegenerative diseases (NDDs) are a heterogeneous group of disorders characterized by the progressive dysfunction of the structure and function of neuronal and glial cells and their networks in the central (CNS) and peripheral nervous system. NDD is an umbrella term for a range of diseases. There is no known way to reverse the progressive degeneration of neurons, and therefore, these diseases are currently considered to be incurable. For a detailed review on the basic mechanism of neurodegeneration, refer to Jellinger et al. 2010 [1].

Medical research has revealed many similarities and common pathways among NDDs [2]:

1. All these diseases are proteinopathies, characterized by one or more specific misfolded proteins [3], such as β-amyloid (Aβ) and tau in Alzheimer’s disease (AD), α-synuclein (α-syn) in Parkinson’s disease (PD), huntingtin in Huntington disease (HD), and the prion protein in prion diseases.
  Amyloid formation is a widespread phenomenon [4]. Abnormal metabolite assemblies may facilitate seeding the proteins to amyloid structures [5]. Amyloid proteins are resistant to proteolysis and form big aggregates, extra- and intracellular bodies (e.g., Lewy bodies, amyloid plaques, neurofibrillary tangles (NFTs)). Proteinopathies are primarily caused by aggregates in different cellular structures (cytosol, endoplasmic reticulum (ER), nucleus) or in the extracellular space. Studies of NDDs (e.g., AD, PD) demonstrated the neurotoxicity (pathogenicity) of amyloid proteins, and thus, the atypical proteins are potential therapeutic targets.
2. Most NDDs have familial (autosomal dominant, inherited) and sporadic forms [6].
3. Many NDDs are late-onset diseases, and therefore, their greatest risk factor is the aging process [7]. In each disease, neurons gradually lose their functions during aging. DNA damage and subsequent deterioration of cellular homeostasis might be a causative link between aging and neuronal loss [8]. Mitochondrial dysfunction increases the level of reactive oxygen species (ROS) that are the main source of DNA damage.
4. Insufficient clearance of the misfolded proteins might also participate in the progress of NDDs. Both the ubiquitin–proteasome and the autophagy–lysosome pathways may contribute to neuronal loss. Proteostasis failure plays a key role in the progress of NDDs [9].
5. Chronic neuroinflammation (elevated and prolonged immune response) is characteristic in NDDs [10,11]. Microglia, astrocytes, endothelial cells, and peripheric immune cells communicate by cytokines and chemokines, causing a prolonged pro-inflammatory state of microglia, opening of the blood–brain barrier and penetration of the immune cells into the CNS [12,13].
6. Dysfunctional mitochondria and altered energy metabolism are also characteristic in NDDs [1,14,15]. The mitochondrial citric acid cycle can regulate the pathogenesis of neuroinflammation [16], and mitochondria represent potential targets in AD therapy [17]. Calcium dyshomeostasis may drastically alter mitochondrial activity, which may drive neurodegeneration [18].
7. Disrupted axonal transport might be one of the greatest problems in the survival of degenerating neurons [1,19].
8. Prion-like self-propagation of amyloids from cell to cell is responsible for the rapid transsynaptic spread of the disease along the anatomical pathways [20].
9. Dysfunction and loss of cells in the CNS cause mild or severe problems with movement (ataxia) and cognitive processes (dementia). Mental abilities generally decline into severe dementia.

These similarities suggest that therapeutic methods and drugs against one NDD might be applied for the treatment of other diseases as well.

Molecular pathological research revealed several differences among NDDs, such as the particular region of the CNS involved in the disease. The molecular pathological classification of NDDs has recently been widely reviewed [21]. Molecular events in the different NDDs may also be distinct [22].

In the present review, we summarize our knowledge on a novel target for the treatment of NDDs, the activation of the sigma-1 receptor (Sig-1R) for the modulation of elevated immune response by inhibiting chronic inflammation in the CNS. This review focuses only on AD, PD, ALS, and HD.

## 2. Neuroinflammation in Different Neurodegenerative Diseases: Cellular Players

Inflammation occurs in the tissues after exposition to microbial pathogens, toxic cellular components, or mechanic tissue injuries. Major immune cells are involved in inflammation (e.g., monocytes, macrophages, T-cells, and B-cells). The innate immune system represents the first line of defense. By now, the importance of neuroinflammation in the progression of NDDs is widely accepted. The recognition that modulation of the immune response contributes to the pathogenesis of NDDs provides many potential targets for treatment.

In AD, the Aβ peptide forms extracellular plaques, and hyperphosphorylated tau forms intracellular deposits (e.g., NFTs) in different brain regions. AD is not one disease, but rather a syndrome [23]. Several disease models have tried to classify AD into different subgroups [24], and the newest probabilistic model proposes the existence of three distinct variants [25]. Neuroinflammation is present in each AD variant and should be included in biological definitions of AD (28) as a third hallmark in addition to β-amyloid deposits and NFTs. Microglia and inflammation have been known targets of AD for several years [26]. Similarly, neuroinflammation has been identified as a potential therapeutic target for neuroprotection in PD [27], ALS [28,29], and HD [30].

The role of inflammation in the development of NDDs has been discussed for a long time, as it has been very difficult to determine whether inflammation is a cause or consequence of neuronal death in CNS (“Correlation is not causation”) [31]. By now, the importance of inflammation in the progress of NDDs has been proven due to epidemiological and genetic evidence. Inflammation has a Janus-faced character. In the beginning, it shows a protective function, a beneficiary effect against many types of acute damage [32]. Activated microglia and astroglia cells of the innate immune system fight against damaging pathogens or tissue injury and prevent infection and cell death.

*Microglia.* Microglial cells are brain-resident phagocytes of the immune system, and they actively maintain brain health. Microglia survey brain regions for phagocytotic clearance of pathogens and cell debris, and they also perform the pruning of synapses [33].

**Figure 1:**
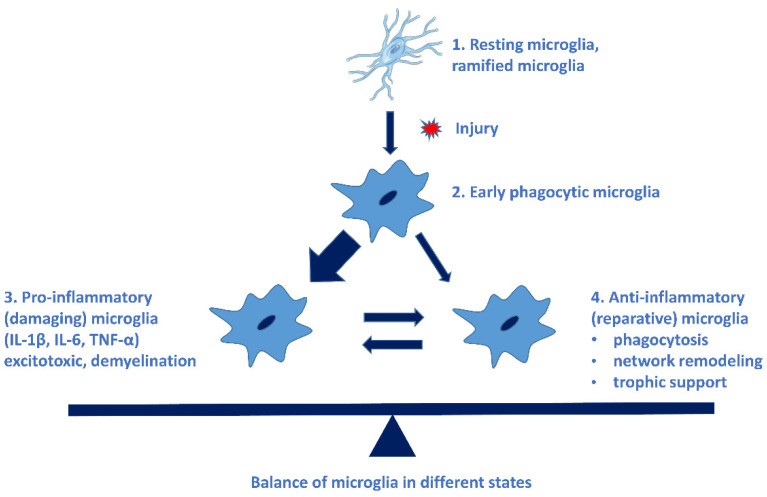
Different types of microglial cells, their characteristic cytokines, and their roles in cellular processes. The imbalance of pro- and anti-inflammatory microglia leads to the progression of NDDs. (See Ref. [31]).

Microglial cells possess extraordinary plasticity and diversity [34], although their classification is not uniform. The following microglial subtypes have been described in AD: plaque-associated (PAM), disease-associated (DAM), and dark microglia and human AD-bound microglia [35]. Resting microglia can be activated in a very short time: within some minutes of the activation, they begin to phagocyte the invader and produce pro- and anti-inflammatory cytokines. These early phagocytic microglia can be rapidly converted to damaging microglia (pro-inflammatory, M-phenotype), which are neurotoxic by producing free radicals and pro-inflammatory cytokines, chemokines. Microglial cells do not return to their resting state, thus causing a chronic inflammation process, and sustained inflammation is detrimental [31]. This state is characterized by a high level of pro-inflammatory cytokines, reduction in neuroprotective factors, dysfunction of the blood–brain barrier (BBB), and penetration of peripheric immune cells into the brain [13]. The neuroprotective (M2 phenotype) microglia have a reparative role in the CNS [31]. Figure 1 demonstrates the types of microglial cells, their characteristic cytokines, and their role in cellular processes [36].

Recently, a novel type of microglia (‘dark’ microglia) with condensed cytoplasm was observed in brain tissues. Dark microglia are hyperactive cells and have dysregulated functions with synapses. Normal aging may also lead to some alterations (senescent microglia: diminished activation, motility, and migration) that decrease the protective function of immune cells [34].

**Figure 2:**
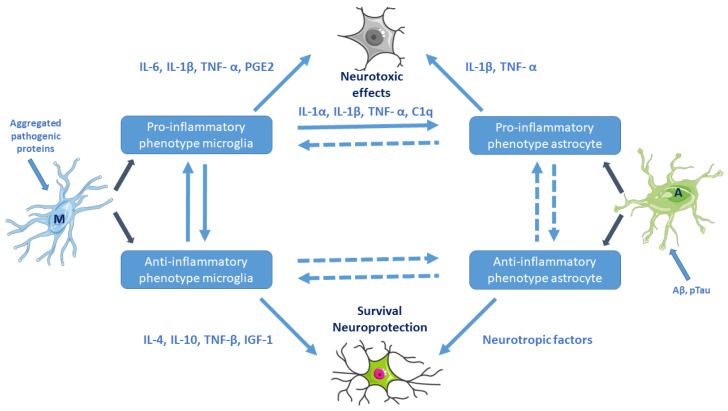
Activation of microglia and astrocytes (e.g., by aggregated pathogenic proteins) and their cross-talk. Pro-inflammatory microglial cells are neurotoxic. Reactive astrocytes can be both beneficial and harmful to surrounding neurons. Microglia–astrocyte cross-talk is maintained by secreted canonical cytokines, growth factors, neurotransmitters, glia-transmitters, and chemokines. M: resting microglia, A: naive astrocyte. (Based on Kwon et al. 2020, Ref. [36]. Cell images from the Servier Medical Art repository were used).

Aβ oligomers and pTau directly activate microglia and astrocytes to produce pro-inflammatory cytokines (Figure 2). Accumulation of misfolded proteins (Aβ, huntingtin, etc.) may result in microglial priming (exaggerated inflammatory response) [34,37]. Microglial priming causes resistance to regulation, impaired response to anti-inflammatory cytokines and collapse of the fine coordination between the immune and nervous system. Microglia-mediated neuroinflammation may play a key role in the progression of NDDs [38]. Increased levels of reactive microglia and pro-inflammatory cytokines (e.g., IL-1β, TNF-α) and chemokines have been found in the brain of AD, PD, and HD patients (Section 3).

*Astrocytes.* Another type of glial cells, the astrocytes, which are the most common and abundant brain cells, also participate in the development of NDDs as key regulators of inflammatory responses. Astrocytes are responsible for brain homeostasis (regulation of blood flow, modulation of the synapse formation, maintenance of the BBB, energy supply for neurons, regulation of the composition of the chemical environment in the extracellular space). Similar to microglia, reactive astrocytes also have pro-inflammatory (A1) and neuroprotective (A2) phenotype subtypes. Astrocytes can also upregulate several genes, thereby activating pro-inflammatory cytokines (IL-1β, TNF-α) [36].

Anti-inflammatory cytokines may trigger neuroprotective astrocytes. The role of astrocytes in the initiation and progression of NDDs is widely accepted [39]. Postmortem studies on AD brains have demonstrated the presence of atrophic astrocytes and a close interaction between astrocytes and amyloid plaques, as well. Astrocytes might be involved in Aβ biosynthesis by upregulating beta-secretase 1. Reactive astrocytes interact with neurons and microglia, thus contributing to the development of AD [40].

Microglia–astrocyte interactions represent a delicate balance affecting neuronal functions in health and disease. Astrocytes–microglia together with Glu-ergic neurons constitute a unit (“quad-particle synapse”, neuro-immune communication). Astrocyte–microglia cross-talk is maintained by secreted canonical cytokines, growth factors, neuro- and glia-transmitters, and chemokines (Figure 2).

*Neuron–glia crosstalk* may play a decisive role in neuroprotection and antioxidant defense mechanisms [41]. The coordinated action of glial cells has been referred to as a “symphony” in the CNS [42]. Both activated microglia and astrocytes participate in the neuroinflammation process [43] (Figure 2).

Two very recent studies analyzed the role of neuroinflammation, microglial activation, and the inflammatory cascade in the pathogenesis of AD and other NDDs (Section 3) [44,45]. Taken together, activated microglia may have different influences on the development and progression of AD, depending on the stage of the disease and the state of microglial priming.

Several studies support the role of the neuroinflammatory response in the development of PD [45], and therefore, the modulation of inflammation is a therapeutic target [46]. Misfolded, toxic α-synuclein (the main amyloid protein in PD) oligomers bind to toll-like receptor 2 (TLR2), which activates inflammatory responses in microglia [47]. A very recent study reviews the role of microglia in neuroinflammation in PD [48].

The role of inflammation in ALS pathogenesis has been widely reviewed [49,50]. Very recent studies demonstrate that microglial activation is also involved in HD development and progression [51]. In 2021, a new mechanism causing nerve destruction in ALS was discovered: TDP-43 protein accumulates and aggregates in axons and neuromuscular junctions to toxic assemblies. These TDP-43 amyloids inhibit the local synthesis of mitochondrial proteins, and thus, disrupt the neuromuscular junctions [52].

## 3. Extracellular Molecular Regulators in Neuroinflammation

Cytokines and chemokines play a key role in the regulation of the recruitment of specific leukocytes during both the acute and chronic inflammation processes [53]. These regulators control complex intracellular signaling mechanisms. This review focuses only on the role of the main cytokines as well as their importance in NDDs. According to very recent literature data, the inflammation cascade plays crucial role in the pathogenesis of AD [43]. One of the main planners of the cascade is the NLRP3 inflammasome.

Several studies demonstrated that insoluble amyloid aggregates (Aβ, tau, α-syn, huntingtin) can trigger inflammatory processes. Soluble Aβ oligomers can also activate the microglia receptors [43].

**Figure 3:**
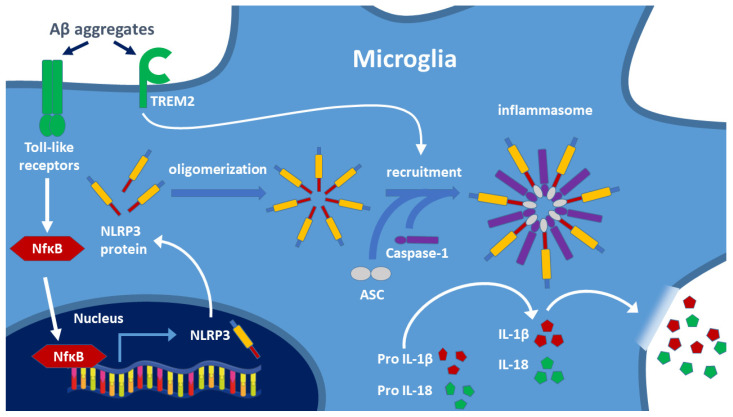
Two signals are involved in the formation of the NLRP3 inflammasome: activation of toll-like receptors (TLR) and TREM2 by toxic amyloid aggregates. Biosynthesis of the NLRP3 protein after NF-κB induced gene expression results in oligomerization of the protein. Recruitment of caspase-1 and ASC generates the NLRP3 inflammasome, and caspase-1 cleaves the IL-precursor proteins leading to the release of mature cytokines IL-1β and IL-18. These cytokines activate the cells of the immune system and trigger the inflammatory response, including the opening of the BBB. TREM2: triggering receptor expressed on myeloid cells 2; ASC: adapter apoptosis-associated speck-like protein containing CARD. (Based on the work of Oliveira et al. 2021, Ref. [43]).

The best-known pathway is the activation of microglial toll-like receptors (TLRs, e.g., TLR-2, TLR-4, TLR-6). This pathway is responsible for the maturation process of IL-1β, a key factor in the pathophysiology of AD. Two signals are necessary for the release of the pro-inflammatory cytokines IL-1β and IL-18. In the first signal, TLR-bound ligands (e.g., LPS, Aβ aggregates) trigger the expression of NLRP3 protein via the nuclear transcription factor NF-κB [43], and then the released NLRP3 monomers may form oligomers (Figure 3). The second signal arises if Aβ binds to the triggering receptor expressed on myeloid cells 2 (TREM2) protein [54] (e.g., if decreased lysosomal degradation results in high Aβ level). It has been demonstrated that TREM2 binds Aβ and modulates microglial function [55,56]. TREM2 causes disruption of lysosomes. Release of cathepsin-B induces the formation of NLRP3 inflammasome assembly containing caspase-1. Caspase 1 cleavage activates the formation of inflammatory cytokines (IL-1β, IL-18) from the precursor proteins, and then, these cytokines activate microglia and other macrophages, thereby inducing inflammation. This pathway has been described for AD; however, most likely it is also valid for other NDDs [56,57,58]. Inflammasome activation leads to apoptosis and pyroptosis of cells (the latter is a less organized form of cell death). Degradation of the inflammasome components is necessary for blocking the inflammatory response and protecting cells. NF-κB, an inducible transcription factor, plays a central role in the regulation of inflammation. NF-κB as a mediator induces the expression of various pro-inflammatory genes as well as the NLRP3 inflammasome (Figure 3).

Longitudinal PET studies have discovered a strong relationship between Aβ and tau amyloid deposition and neuroinflammation [59]. Very recent studies have shown that tau protein (the other key player in the progression of AD) interacts with the intracellular polyglutamine binding protein 1 (PQBP1), and thereby, it activates pro-inflammatory genes that induce inflammation [60].

In a mouse model of AD, the R47H-TREM2 mutation induced NDD by robustly increasing pro-inflammatory cytokines via hyperactivation of AKT signalization [61].

Although IL-1β and IL-18 are very important in NDDs, other inflammatory cytokines (e.g., IL-17) are also among the key players. The IL-17 cytokine family contains several pleiotropic inflammatory molecules. There has been a long debate on the role of the cytokine IL-17A in the progression of AD. IL-17A is produced by T helper 17 (Th 17) cells and has multifaceted roles [62]. Several authors state that IL-17A represents the key cytokine in chronic inflammatory NDDs [63,64]. Neutralization of IL-17 with a specific antibody rescues Aβ-induced inflammation and memory impairment in mice models [65,66]. IL-17A and its receptor may serve as a checkpoint in microglia-mediated neuroinflammation since their blockade was shown to protect neurons in mice [67]. According to recent results, IL-17A causes inflammation and plays an important role in olfactory impairment, cognitive dysfunction, Aβ-accumulation, tau-hyperphosphorylation, BBB leakage, neuronal loss, neurogenesis, and synaptic plasticity [68]. IL-17A seems to be a potential and sensitive biomarker and an important pharmacological target of AD.

Other factors, e.g., chemokines, also play a key role in the regulation of microglial migration in the CNS [69].

The complement system may also participate in the neuroinflammation; however, its role has not yet been clarified in NDDs.

## 4. Therapeutic Use of Classical Anti-Inflammatory Drugs in NDDs

The role of neuroinflammation in the development of NDDs has been widely accepted due to epidemiological, neuroimaging, and genetic evidence. As a consequence, it has been expected that anti-inflammatory therapies would be beneficial in the prevention and treatment of NDDs. Epidemiological studies suggest that anti-inflammatory drugs (e.g., aspirin and the classical non-steroidal anti-inflammatory drugs, NSAIDs) could be helpful in preventing AD; however, the administration of these drugs has been ineffective in clinical trials with AD patients. The outcome of the long trial ADAPT (Anti-inflammatory Prevention Trial), a randomized placebo-controlled study using naproxen and celecoxib, had negative results. A 2009 review of the results of the efficacy of NSAIDS in AD therapy found that chronic use of NSAIDs in AD patients proved to be effective only before the Aβ deposition started [70]. The results of targeting neuroinflammation in AD were reviewed in 2016 [71] and 2017 [72]. The use of NSAIDS for AD prevention proved to be unsuccessful: two years of daily naproxen administration to people in their sixties with a high risk of AD caused several side effects and non-beneficial events, and unexpectedly promoted AD progression [73]. The conflicting results and opinions on the use of NSAIDs for the prevention of AD have been analyzed and reviewed recently [74]. The final conclusion of this review was that in diagnosed AD cases the use of NSAIDs is currently not recommended, as randomized control trials (RCTs) failed to prove their benefits. The literature review also points out the discrepancies between the results of observational studies and RCTs. Recent studies of P. McGeer [75] have been heavily criticized, pointing out that daily ibuprofen causes harmful interactions and increases the risk of stomach ulcer and intestinal bleeding (ibid, D. Brown). A very recent review thoroughly analyzed the results of the clinical trials with NSAIDs in AD treatment [76] and found that the use of NSAIDs was associated with decreased AD prevalence; however, the review observed no beneficial effect on cognitive decline. The latest analysis of the past clinical trials with NSAIDs has suggested that drug failures may result from various mistakes in the planning of trial protocols—for example, the treatment timing was too short (6–12 months), the patients were too old or severely ill, or the trial groups were genetically inhomogeneous [77]. New trials should be performed using both longer and earlier drug interventions and better criteria for the selection of patients.

It is very important to remember that a large cohort of epidemiological studies showed that the use of NSAIDs was associated with a lower risk of AD development. New genetic evidence (e.g., the role of TREM2 mutations in AD) unequivocally show the causal role of innate immunity in AD risk. Novel clinical studies may solve the problem of conflicts between epidemiological, experimental, and clinical results [77].

NSAIDs have also been used for the treatment of PD, as compelling evidence showed the contribution of neuroinflammation to the pathogenesis of PD [78]. The use of NSAIDs in PD gave conflicting results [79].

An FDA-approved anti-inflammatory drug (cromolyn sodium) can delay the symptoms of ALS development in a mouse model of the disease [80]. Another meta-analysis resulted in conflicting results: non-aspirin-NSAIDs and acetaminophen were associated with an increased risk of ALS progression; however, aspirin did not affect the development of disease [81].

Immunotherapies and anti-inflammatory agents have not yet shown effective neuroprotection in clinical trials with HD patients [82]. Targeted multimodal therapy against inflammation shows promise in slowing down HD progression.

## 5. Sig-1 Receptor as a Novel Drug Target for Neuroprotection

### 5.1. Molecular Function of the Sig-1R, a Ligand-Operated Chaperone

The molecular role of Sig-1R has been studied for over 40 years. It has been revealed that Sig-1R plays an important role in the protection of neurons. The structure of Sig-1R is not similar to any known receptors or mammalian proteins. To date, no endogenous ligand has been found for Sig-1R. A very large number of proteins interact with Sig-1R, which acts as a modulator of cellular signaling. It is widely accepted that Sig-1R is a ligand-operated chaperone [83]. Signalization pathways during ER (endoplasmic reticulum) stress are coupled to the activation of Sig-1R, and this receptor plays a key role in cellular stress signaling [84]. By regulating ER stress, Sig-1R influences the signaling of a number of cellular pathways without direct association with other proteins.

The physiological role and the molecular functions of Sig-1R have been recently reviewed [85,86,87]. Sig-1R is a non-opioid, non-G-protein coupled, non-ionotropic, ligand-operated intracellular chaperone that is located in the mitochondrial-associated membrane part (MAM) of the ER membrane. As a chaperone, Sig-1R interacts with over 50 proteins and integrates a lot of signaling pathways [84]. In particular, it modulates Ca^2+^-signaling via the inositol triphosphate receptor (IP3) on the ER. Sig-1R can translocate within the cell to the plasma membrane, where it interacts with ion channels [88]. Pathways by which Sig-1R regulates ER-stress and calcium homeostasis, as well as oxidative stress, have been discovered and validated [89]. According to the latest results, Sig-1R is at the crossroad of proteostasis, neurodegeneration, and autophagy [90]—therefore, a central regulator of cell survival.

Sig-1R is highly expressed in the CNS where it possesses very important functions (cell differentiation, formation of axons, synaptic growth, activation of microglia, and regulation of astrocytes) [89]. It regulates neuroprotective mechanisms, e.g., it promotes the expression of BDNF and nerve growth factor (NGF) and also supports neuronal repair. Activation of Sig-1R stimulates brain plasticity and prevents the deterioration of the BBB. Sig-1R stimulation plays a decisive role in the inhibition of ER stress, Ca^2+^ toxicity, and the inflammatory response.

### 5.2. Sig-1R as a Central Regulator of Cell Survival (ER-Stress, Autophagy, Inflammation)

**Figure 4:**
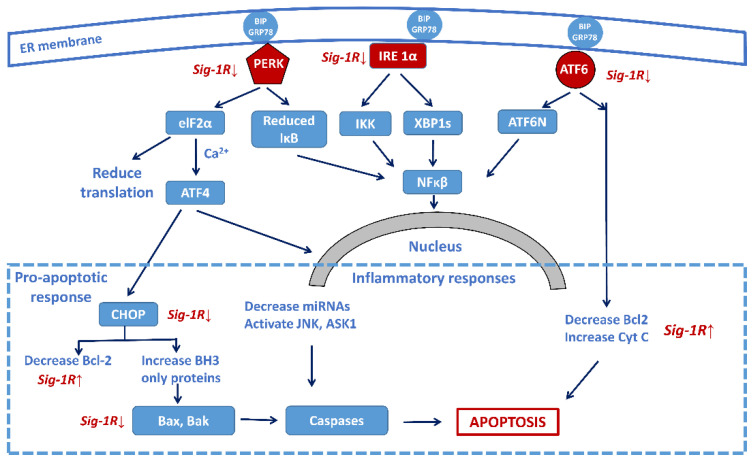
Sig-1R regulation of the three main signaling pathways of ER stress-activated unfolded protein response (UPR) focusing on the central role of the transcription factor NF-κB. Sig-1R activation increases cell survival by the attenuation of the activity of the three sensors (PERK, IRE-1α, and ATF6) and decreasing the pro-apoptotic responses, as well as increasing the anti-apoptotic Bcl-2 activity. (Abbreviations: PERK: protein kinase RNA-like ER-kinase; IRE1α: inositol requiring enzyme 1α; ATF6: activating transcription factor 6; eIF2α: eukaryotic translation initiation factor 2α; XBP1: X-box binding protein 1 (spliced form); TRAF2: TNF-associated factor-2; ATF4: transcriptional activator factor-4; MT: mitochondrion; CHOP: c/EBF homologous protein; Bcl-2: B-cell lymphoma 2; Bax: Bcl-2 like protein 2; Bak: Bcl-2 homologous antagonist killer; JNK: c-Jun terminal amino kinase; ASK1: apoptosis signal-regulating kinase).

Persistent ER stress may drive the pathology of many chronic disorders, including NDDs [91]. Accumulation of misfolded proteins in brain cells (a hallmark of NDD pathology) induces a highly conserved stress response, the unfolded protein response (UPR), for maintaining homeostasis [92]. UPR has three phases. In the adaptive phase, cells synthesize a higher amount of chaperone proteins for protecting themselves from the effects of misfolded proteins. In a more severe situation, the compensatory mechanisms of UPR start if the amount of wrong protein structures overwhelms the folding capacity of ER. An elevated level of misfolded proteins alarms the extracellular environment by activating the inflammatory pathways (pro-inflammatory phase). When all these adaptive mechanisms fail, the UPR triggers cell death in a caspase-dependent or independent way (pro-apoptotic phase) [93]. Figure 4 shows the three major signalization pathways with the three sensor molecules that mediate UPR: the IRE1α, the PERK, and the ATF6 pathways.

UPR represents a bifunctional response of the cells to protein misfolding with both pro- and anti-survival effects. Basal activity of the UPR is beneficial for cell survival: activation of ER-assisted degradation (ERAD) may clear the toxic misfolded proteins (cell maintenance program). However, continuous ER stress and chronic UPR may trigger cell death. The early events and steps of UPR leading to changes in gene expression have been recently reviewed [94]. ER stress induces inflammation via NOD1 and NOD2 (nucleotide binding oligomerization domain 1 and 2) signaling [95]. ER stress also activates the NLRP3 inflammasome [96]. Recent data indicate that the UPR and NF-κB pathways converge in the cell nucleus via ten major transcription factors [97]. The occupancy of the enhancer and promoter regions by these factors coordinates the activity of hundreds of genes and determines the balance between apoptosis and repair of cell damage and survival. ER stress is associated with the aging process and is a potent inducer of inflammation [98]. Signalization pathways during ER stress are coupled to the activation of the Sig-1R that plays a key role in cellular stress signaling [84].

Recent reviews demonstrate the role of Sig-1R in NDDs [99,100,101]. The receptor has been a target for treating ALS, PD, HD, and AD [102]. Sig-1R regulates ER stress via the three sensor molecules shown in Figure 4. Sig-1R agonists have shown a cytoprotective effect in stroke, and this effect is associated with reduced ER stress [103]. Activation of Sig-1R elicits very effective neuroprotective processes and promotes neuronal survival [104]. In an inflammation and sepsis model experiment, the modulation of the Sig-1R–IRE1 pathway with the agonist fluvoxamine proved to be beneficial [105].

All experimental data so far suggest that activation of Sig-1R improves NDDs by balancing ion homeostasis, regulating ER (and oxidative) stress, promoting the expression of neurotrophic factors, and helping nerve remodeling. Based on these neuroprotective effects, Sig-1R has become one of the most important targets in drug research for the treatment of NDDs [89].

### 5.3. Sig-1R Ligands in the Therapeutic Treatment of NDDs

Tremendous scientific work has been performed for NDD drug research; however, no drugs with a curative effect have been found. In 2021, PhARMA released a new detailed report on more than 260 potential medicines for the treatment of 29 different NDDs, all of which are in clinical trials or awaiting review by the U.S. FDA. Among these potential and investigational drugs, 85 are in development for AD, 64 for PD, 38 for ALS, and 14 for HD [106]. Several FDA-approved former drugs have been repurposed for using them in neuroprotection (e.g., the NKCC1 chloride channel modulator bumetanide for treating APO E4-related AD [107]).

Many of the latest compounds in the pipeline are Sig-1R ligands. Neuroinflammation has an impact on adult neurogenesis, and both processes might be potential targets for treating NDDs [108]. During the last 10 years, pridopidine has been at the center of preclinical research and clinical trials of NDDs. The compound was originally developed for the treatment of motor symptoms of HD. Pridopidine (a low-affinity dopamine receptor D2 antagonist) is an FDA-approved investigational drug as a dopamine stabilizer. However, it also has a high affinity towards Sig-1R with a K_i_ of 70 nM, and it reaches a near-complete Sig-1R occupancy by 90 mg/day dose. (Interestingly, it shows minimal D2/D3 receptor occupancy [109]).

Pridopidine was first used in a mouse model of HD, which showed that Sig-1R activation resulted in beneficial effects [110,111,112,113]. Pridopidine normalizes Ca-balance in striatal neurons in HD [114].

The latest studies demonstrate that pridopidine reduces ER stress and rescues mitochondrial function by counterbalancing the toxic effect of huntingtin aggregates by modulating Sig-1R [115,116]. Pridopidine was applied in a PD mouse model, where it induced functional neurorestoration via activation of Sig-1R [117]. Pridopidine was successfully tested for the amelioration of ALS pathology in the SOD1 (D93A) mouse model [118]. Pridopidine also has a neuroprotective effect in cellular and animal models of AD by Sig-1R-mediated stabilization of mushroom-shaped memory spines [102]. The mechanism of action of pridopidine has been identified: the drug increased spine density and long-term potentiation in neurons in vitro and in an AD mouse model. These beneficial effects were prevented with co-administration of siRNA against Sig-1R. Basal activity of Sig-1R is required for mature spine stability, whereas agonist-mediated receptor activity is needed for the stabilization of mushroom spines. Pridopidine improves BDNF and GDNF axonal transport, reduces the level of toxic protein aggregates, restores synaptic activity in neuromuscular junctions, and increases neuronal survival in vivo.

The 2019 Report of the Alzheimer’s Drug Discovery Foundation summarizes the results of preclinical studies with pridopidine in AD, PD, ALS, and HD. Pridopidine proved to have beneficial effects in multiple disease models, though its effectiveness in AD patients is unclear. Human research suggests benefits to patients with dementia and also with HD, although its effects in HD are not consistent.

The last Alzforum Therapeutics (12 October 2021) excellently summarizes the results of the last 10 years of research with pridopidine. The drug is currently in the late stage of clinical development to treat HD (Phase 3) and ALS (Phase 2/3) in multicenter trials, and these trials are expected to be complete by 2022. FDA granted pridopidine an orphan drug status in July 2021.

The second leading Sig-1R agonist under NDD drug development is ANAVEX2-73, a mixed Sig-1R /muscarinic receptor agonist [119]. Several clinical trials show the effect of ANAVEX2-73 treatment on patients with AD and demented patients with PD. Multivariable analysis of a completed phase 2a trial in patients with AD showed improved responses after a one-year administration of high doses (30 and 50 mg) of ANAVEX2-73 (Anavex Life Sci. Corp. data published in October 2017). In August 2018, the firm started a phase 2b/3 clinical trial for the treatment of early AD. Studies in an AD mouse model showed that the drug can be combined with donepezil for increasing the neuroprotective effect [120].

Another Anavex compound, AF710B (ANAVEX 3-71), is also a mixed Sig-1R/muscarinic M1 agonist [121]. AF710B showed dose-dependent therapeutic efficacy in AD animal models.

*Fluvoxamine*, a selective serotonin reuptake inhibitor (SSRI) with Sig-1R agonist activity (K_i_ = 36 nM) may be repurposed for treating dementia. Fluvoxamine enhances cellular resistance to ER stress and decreases tau phosphorylation [122]. The drug also decreases Aβ production. The reduction in the Aβ level [123] and Sig-1R activation are responsible for the decreased Aβ biosynthesis.

*Citalopram* is also an SSRI with Sig-1R agonist affinity (K_i_ = 292 nM). The drug decreased the formation of new amyloid plaques in an AD mouse model [124]. In healthy humans, citalopram administration decreased Aβ concentration in the CSF. Citalopram also demonstrated other benefits in dementia [125]. 

*Dextrometorphan* is a noncompetitive NMDA antagonist and a non-selective Sig-1R agonist. Quinidine extends the effect of dextromethorphan, and the combination of the two drugs was approved by the FDA under the name of AVP-923 for the treatment of ALS. AVP-293 was successfully used in a clinical phase 2a trial with AD patients [126].

*Donepezil (Aricept)*, an acetylcholinesterase inhibitor used for symptomatic treatment of AD, is also a Sig-1R agonist [127]. It has been well known that many currently marketed drugs (e.g., haloperidol, fluvoxamine, and also donepezil) interact with Sig-1R, although not selectively [99]. PET studies demonstrated that donepezil binds to Sig-1R in the living human brain with a high-binding site occupancy.

## 6. Conclusions, Outlook

Chronic neuroinflammation is a hallmark of NDDs (AD, PD, ALS, and HD). Genetic evidence supports the theory that anti-inflammation therapy could be used for curing these diseases. Although epidemiological studies support the neuroprotective action of NSAIDs in the prevention of NDDs, anti-inflammatory clinical trials with NSAIDs have been unsuccessful.

As Sig-1R controls ER stress and the inflammatory processes in the cells, Sig-1R agonists have been tested in preclinical studies and clinical trials for neuroprotection in NDDs.

Many currently marketed drugs interact with Sig-1R and behave as agonists but are not selective (promiscuous ligands). Their neuroprotective action has been demonstrated in animal models of NDDs. Several clinical trials are currently ongoing with these drugs and their combinations. Use of drug synergism and targeted multimodality therapy are promising methods for the future. Repurposing some FDA-approved drugs for activation of Sig-1R can also result in rapid development of new neuroprotective agents from old drugs.

## Data Availability

Not applicable.
